# Efficacy of titanium locking plate combined with suture anchor repair on ankle joint functional recovery and quality of life in patients with ankle fractures

**DOI:** 10.12669/pjms.40.10.10308

**Published:** 2024-11

**Authors:** Zhenfeng Huang, Mengni Chen, Zhiwei Ye

**Affiliations:** 1 Zhenfeng Huang Department of Traumatology and Orthopedics, Wuhan Fourth Hospital, Wuhan, Hubei Province 430000, P.R. China; 2 Mengni Chen Department of Operating Room, Tongji Hospital Affiliated to Tongji Medical, College of Huazhong University of Science & Technology, Wuhan, Hubei Province 430030, P.R. China; 3 Zhiwei Ye Department of Traumatology and Orthopedics, Wuhan Fourth Hospital, Wuhan, Hubei Province 430000, P.R. China

**Keywords:** Ankle fractures, Locking titanium plate, Suture anchor repair, Generic Quality of Life Inventory-74 (GQOL-74)

## Abstract

**Objective:**

To evaluate the efficacy of titanium locking plate combined with suture anchor (SA) repair in the treatment of ankle fractures (AF).

**Methods:**

Retrospective analysis of clinical data of 170 patients with AF who underwent surgical treatment at the Department of Traumatology and Orthopedics of Wuhan Fourth Hospital, China from May 2020 to June 2023 was conducted. A total of 83 patients underwent conventional open reduction and internal fixation (control group); 87 patients received titanium locking plates combined with SA repair (observation group). Ankle joint functional recovery (measured using the Mazur Ankle Grading Scale), quality of life (measured using the Generic Quality of Life Inventory-74), and incidence of complications were compared between two groups. Active range of motion and muscle strength of the ankle joint in plantar flexion and dorsiflexion states, and medial clear space and talar tilt angle were also analyzed.

**Results:**

The rate of excellent recovery of ankle joint function in the observation group was significantly higher than that in the control group (*P*<0.05). Six months after surgery, active range of motion and muscle strength of the ankle joints in the plantar flexion and dorsiflexion states in the observation group was significantly higher than that in the control group (*P*<0.05). The medial clear space and talar tilt angle of the observation group was significantly smaller compared to the control group (*P*<0.05). The quality of life in the observation group was significantly higher than that in the control group, and the incidence of complications in the observation group was significantly lower than that in the control group (*P*<0.05).

**Conclusions:**

Titanium locking plates combined with SA repair for the treatment of AF can significantly improve ankle joint functional recovery, improve the quality of life of patients, and is associated with a lower incidence of complications.

## INTRODUCTION

Ankle joint is an important load-bearing joint in the body that is particularly prone to fractures under external force due to its unique anatomical structure.^1^ AF are often accompanied by damage to the surrounding tissues, which may have a serious impact on the overall stability of the body. Additionally, reduced contact surface of tibia may lead to local stress accumulation in the foot and cause serious adverse reactions including a degenerative joint disease.^2^,^3^ If patients with AF do not receive timely and effective intervention, they may develop traumatic arthritis, joint pain, and ankylosis, associated with a serious impact on the daily activities and quality of life of patients.^4^,^5^ Therefore, implementing safe and effective treatment measures for patients with AF is crucial.

The use of titanium locking plates is considered an important component of the orthopedic surgery for AF, as it can restore local anatomical structure of the joint, accelerate fracture healing, and facilitate the recovery of ankle joint function.^6^ In the past, the main focus of the AF repair surgery was on the anatomical reduction of the ankle joint bone structure, while insufficient attention was paid to the injury and repair of the medial ankle deltoid ligament. The triangular ligament is an important stable structure on the medial side of the ankle joint, and when damaged, is associated with a high incidence of joint instability and traumatic arthritis, which is not conducive to good rehabilitation of ankle function.^7^,^8^

Arthroscopic repair of the triangular ligament with SA is increasingly used in AF surgery, and the thread rivet, a special titanium nail with strong retention force, can be completely embedded in bone tissue with minimal trauma, achieving good results.^9^,^10^ At present, there is limited literature on the use of titanium locking plates combined with SA repair for AF. This study aimed to evaluate clinical intervention effect of titanium locking plates combined with SA repair in patients with AF.

## METHODS

Clinical data of 170 patients (93 males and 77 females) with AF who underwent surgical treatment at the Department of Traumatology and Orthopedics of Wuhan Fourth Hospital, China from May 2020 to June 2023 were retrospectively analyzed. Age of the patients ranged from 22 to 75 years, with an average of 49.55 ± 12.58 years. Of 170 patients, 83 underwent conventional open reduction and internal fixation treatment, and were set as the control group, and 87 were operated using titanium locking plates combined with SA repair and designated as the observation group.

### Ethical approval:

The ethics committee of our hospital approved this study, No. KY2024-008-01, Date: February 1, 2024.

### Inclusion criteria:

- Patients diagnosed with AF requiring ligament repair through CT, X-ray or MRI.
- Patients underwent surgery for the first time.
- Fresh closed AF.
- Having a clear cause of injury.
- The clinical data was complete.

### Exclusion criteria:

- Patients with open fractures.
- Patients with vascular/nerve injury.
- Patients with pathological fractures.
- Patients with other combined fractures.
- Female patients during lactation/pregnancy.
- Patients with osteoporosis.
- Patients with combined bone tumors and bone tuberculosis.

### Routine open reduction and internal fixation treatment:

Computer tomography (CT) was performed to clarify the status of fractures around the ankle joint. Under general anesthesia, patient was placed in a lateral position, the affected limb was elevated, and an inflatable tourniquet was applied to the thigh root. Prophylactic administration of antibiotics was done during skin cutting. Fracture reduction was done according to the following sequence: external ankle, internal ankle, and posterior ankle. Outer ankle was opened and exposed, and the fractured end was reduced. The pre molded distal fibular steel plate was fixed on the outer side of the fibula, and the drill bit was changed to a coarse when using a screw channel.

Kirschner wire was used to preserve bone mass to the greatest extent possible. A curved incision was made on the inner ankle (with protection of the great saphenous vein, and cleaning of soft tissue at the fracture site). Under direct visualization, the ankle joint was reduced and fixed. Two Kirschner wires were positioned vertically along the fracture line, and the triangular ligament injury was repaired with an absorption line. The stability of the tibia and fibula was determined through C-arm X-ray examination. If there was a lack of stability, the lower tibia and fibula were tightened with reduction forceps, and the anterior tibiofibular joint and ligament of the lateral ankle and tibia were fixed with screws (ensuring that the screws penetrate through two layers of fibular cortex and one layer of tibia). The affected limb was then fixed with plaster after the surgery.

### Titanium locking plate combined with SA repair:

Patients were instructed to lie flat and general anesthesia was initiated. A surgical incision was made outside the ankle joint to effectively expose the posterior ankle, the fracture end was fixed with a locked titanium plate and the posterior ankle was internally fixed with hollow screws. Lower tibiofibular joint was fixed with tibiofibular screws from the posterior lateral side of the fibula to the anterior medial side of the tibia, ensuring satisfactory reduction of the lower tibiofibular joint, and the screws were tightened to repair the lower tibiofibular ligament. An arc-shaped incision was made on the inner side of the ankle joint to traction the tendon.

The condition of superficial and deep injuries to the triangular ligament was assessed. If the rupture of the triangular ligament occurred at the starting point of the medial malleolus, the attachment site of the talus, and the body, suture rivets were used to reconstruct and repair the damaged triangular ligament. For patients with talus avulsion at the insertion point, a hole was drilled at the attachment point of the talus, rivets were inserted, and the inner ankle was sutured using the suture pad provided by the rivets. Suture treatment was performed on the broken end of the ankle ligament, with a knot tied in an inverted position, followed by layer by layer closure of the joint capsule and triangular ligament (shallow suture). The stress of the triangular ligament was measured with the assistance of fluoroscopy. After surgery, the ankle joint was fixed with plaster (Supplementary Fig.1).

**Fig.1:**
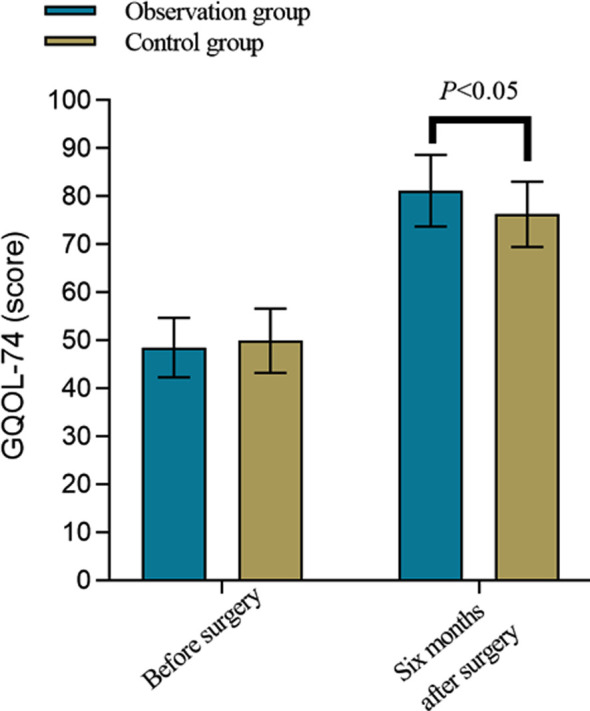
Comparison of GQOL-74 score between Two Groups

**Figure:**
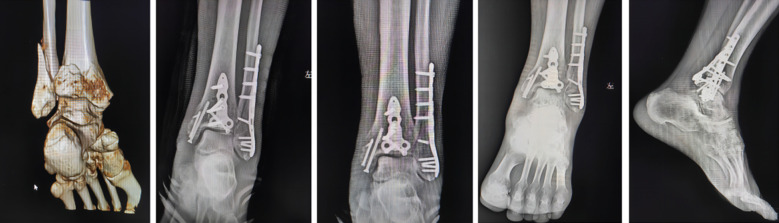
Supplementary Fig.1

### Observation indicators:

- Ankle joint functional recovery was evaluated using the Mazur Ankle Grading Scale, with a total of 100 points. Recovery was categorized as excellent (>92 points), good (87-92 points), general (65-86 points), and poor (<65 points). Excellent and good recovery were included in the “recovery excellent” rate.
- Active range of motion and muscle strength of the ankle joint in plantar flexion and dorsiflexion states.
- Medial clear space and talar tilt angle were measured using X ray and CT from front view before surgery and six months after surgery.
- Quality of life, according to the GQOL-74 comprehensive quality of life assessment questionnaire, with the score of 100 points. Higher the score corresponded to better quality of life.
- The occurrence of complications such as internal fixation fracture, nerve injury, traumatic arthritis, and poor healing.

### Statistical Analysis:

All data analyses were conducted using SPSS 25.0 (IBM Corp, Armonk, NY, USA) and PRISM 8.0 software (GraphPad, San Diego, USA). Quantitative data were represented by mean ± standard deviation, independent sample t-test was used for inter group comparison, and paired t-test was used for intra group before and after comparison. Count data was analyzed using chi square test to represent the number of use cases. P<0.05, was statistically significant.

## RESULTS

A total of 170 patients met the conditions for this study, with 83 patients in the control group, and 87 patients in the observation group. There was no significant difference in general information between the two groups (*P*>0.05), Table-I. The excellent recovery rate of ankle joint function in the observation group was significantly higher than that in the control group (*P*<0.05), Table-II.

**Table-I:** Comparison of baseline data between two groups.

Item	Observation group (n=87)	Control group (n=83)	t/χ^2^	P
Gender [n (%)]				
Male	43 (49.43)	50 (61.45)	2.005	0.157
Female	44 (50.57)	33 (38.55)		
Age (year)	50.24±12.37	48.82±12.84	0.735	0.463
BMI (kg/m^2^)	23.93±2.95	23.23±3.15	1.504	0.134
Cause of fracture [n (%)]			0.593	0.743
Falling from heights	22 (25.29)	18 (21.69)		
Traffic accident	25 (28.73)	28 (33.73)		
Sprain	40 (45.98)	37 (44.58)		
AO classification [n (%)]			3.366	0.186
Type A	20 (22.99)	15 (18.07)		
Type B	52 (59.77)	44 (53.01)		
Type C	15 (17.24)	24 (28.92)		

**Table-II:** Comparison of ankle joint functional recovery between two groups.

Group	n	Excellent	Good	General	Poor	Excellent rate
Observation group	*87*	59 (67.82)	23 (26.44)	5 (5.75)	0 (0.00)	82 (94.25)
Control group	*83*	47 (56.63)	23 (27.71)	11 (13.25)	2 (2.41)	70 (84.34)
*χ* ^2^						4.411
*P*						0.036

Before the surgery, there was no significant difference in the range of active motion and muscle strength of ankle joints in plantar flexion and dorsiflexion states between the two groups (*P*>0.05). Six months after the surgery, active range of motion and muscle strength of ankle joints in plantar flexion and dorsiflexion states significantly increased compared to preoperative levels, and was significantly larger in the observation group compared to the control group (*P*<0.05), Table-III.

**Table-III:** Comparison of active range of motion and muscle strength of ankle joints in plantar flexion and dorsiflexion states.

Time	Group	n	Plantar flexion		Dorsiflexion	

			Active range of joint motion (°)	Muscle strength (N)	Active range of joint motion (°)	Muscle strength (N)
Before surgery	Observation group	87	12.80±2.99	3.12±0.42	9.98±2.09	3.51±0.41
	Control group	83	12.30±3.10	3.07±0.48	10.10±2.42	3.44±0.44
	*t*		1.077	0.769	-0.345	1.111
	*P*		0.283	0.443	0.730	0.268
Six months after surgery	Observation group	87	42.47±6.12^a^	4.81±0.51^a^	28.59±4.20^a^	4.89±0.47^a^
	Control group	83	37.59±7.86^a^	4.37±0.44^a^	23.95±4.73^a^	4.53±0.43^a^
	*t*		4.505	6.019	6.763	5.187
	*P*		<0.001	<0.001	<0.001	<0.001

***Note:*** Compared with the same group before surgery,

a P<0.05.

Before the surgery, there was no significant difference (*P*>0.05) in the medial clear space and talar tilt angle between the two groups. Six months after the surgery, these indices were significantly reduced compared to pre-surgery values, and were significantly smaller in the observation group compared to the control group (*P*<0.05), Table-IV.

**Table-IV:** Comparison of medial clear space and talar tilt angle between two groups.

Time	Group	n	Medial clear space (mm)	Talar tilt angle (°)
Before surgery	Observation group	87	6.65±1.18	12.08±2.90
	Control group	83	6.52±1.36	11.70±2.99
	t		0.657	0.846
	P		0.512	0.399
Six months after surgery	Observation group	87	2.01±0.61	4.44±1.33
	Control group	83	2.56±0.65	5.24±1.74
	t		-5.729	-3.403
	P		<0.001	0.001

***Note:*** Compared with the same group before surgery, ^a^P<0.05.

Before the surgery, there was no significant difference in GQOL-74 scores between the two groups (*P*>0.05). Six months after surgery, GQOL-74 scores in both groups significantly increased, and were significantly higher in the observation group than in the control group (*P*<0.05) (Fig.1). The incidence of complications in the observation group was significantly lower than in the control group (*P*<0.05), Table-V.

**Table-V:** Comparison of complications between two groups.

Group	n	Internal fixation fracture	Nerve injury	Traumatic arthritis	Poor healing	Total incidence rate
Observation group	87	0 (0.00)	1 (1.15)	1 (1.15)	0 (0.00)	2 (2.30)
Control group	83	1 (1.20)	3 (3.61)	3 (3.61)	2 (2.41)	9 (10.84)
*χ* ^2^						5.190
*P*						0.023

## DISCUSSION

The results of this study showed that compared to conventional open reduction and internal fixation treatment, the combination of titanium locking plates and SA repair can more effectively improve the active range of motion and muscle strength of the ankle joint in plantar flexion and dorsiflexion states, and reduce the medial clear space and talar tilt angle, improving the rehabilitation of ankle joint function and helping to reduce the occurrence of complications. This surgical method, therefore, may improve the effectiveness and safety of AF repair.

Our results confirm previous observations. Zhang et al.^11^ who combined open reduction and internal fixation with SA repair for the treatment of AF with triangular ligament rupture in patients, showed that it can effectively improve the effectiveness of disease treatment and reduce the degree of pain. Additionally, the American Orthopedic Association of Foot and Ankle (AOFAS) score were higher in the observation group than in the control group patients who did not receive suture rivet repair treatment. Rigby et al.^12^ demonstrated that SA repair and titanium implants with a tail line at the end can reconstruct the anatomical structure of the triangular ligament through multi-point fixation, thereby restoring the biomechanical relationship and stability of the ankle joint and avoiding long-term postoperative pain.

At the same time, suture strength at the tail end of the threaded rivet is relatively high, which helps to increase the bone tendon fit, accelerate fracture healing process, and improve the rehabilitation effect of ankle joint function. Park et al.^13^ also showed that combined internal fixation treatment and threaded rivet repair can help improve the rehabilitation effect of ankle joint function, alleviate pain, narrow the medial clear space and talar tilt angle, which is consistent with the conclusions of our study.

Previous studies have also pointed out that the balance between the tibia, fibula, and talus is lost after AF, and open reduction and internal fixation can achieve anatomical reduction at the fracture site.^14^,^15^ Triangular ligament plays an important role in maintaining the position of the talus,^16^ and, if damaged, can cause relaxation of blood vessels and corresponding nerves, reduce bone blood supply, and affect bone healing and functional recovery.^15^,^16^ The suture rivet can effectively repair the triangular ligament, and the tail line of the suture rivet has high strength, requiring only a small amount of soft tissue peeling to complete the operation. The effect is significant and the safety is high.^11^-^13^ Luo et al.^17^ also explored the application value of SA repair and internal fixation repair in patients with AF and medial mixed injuries, and confirmed that it can increase the range of motion of ankle dorsiflexion and plantar flexion, improve ankle function, and alleviate pain. Our study is in agreement with these observations.

Studies have confirmed that the procedure of SA repair is simple, and the placement position can be flexibly adjusted according to the patient’s specific condition and treatment plan. The tail end suture sutures the ligament, which can be fixed at multiple points and has strong stability, which is conducive to reducing the risk of internal fixation loosening. Moreover, when SA is done during intraoperative repair, there is a reduction in soft tissue detachment, which can reduce surgical trauma. In addition, there is no need to remove rivets after the surgery, which is conducive to early recovery.^7^,^18^ A study of Xiao et al.^19^ also confirmed that compared to ligament suture screw fixation, SA repair can effectively restore anatomical structure, provide strong internal fixation in a timely manner, and restore the stability of the medial aspect of the ankle joint.

Ligament repair combined with screw fixation is prone to malunion after removing the lower tibiofibular screw. Hong et al.^20^ found that fixing the lateral ankle and lower tibiofibular joint alone makes achieving satisfactory results more challenging, while repairing triangular ligament injuries can improve rehabilitation outcomes. Additionally, SA repair can easily fix the broken end of ligaments to the talus or medial malleolus, with minimal trauma, and completely embed rivets into bone tissue, achieving reliable and firm internal fixation effects. Strength of the suture with rivets is relatively high, and sutures can be closely connected to the bone, making it convenient for early postoperative functional rehabilitation training.^19^-^21^

Our results also demonstrated that the GQOL-74 score of the observation group was higher than that of the control group six months after the surgery. This indicates that the combination of titanium locking plates and SA repair has a significant advantage in improving the quality of life of patients with AF. We may speculate that the combination of titanium locking plates and SA repair can more effectively improve limb function in patients with AF, reduce the occurrence of complications, and alleviate the negative impact of the disease on daily life, manifesting in the significant improvement of patient’s quality of life.^15^,^16^,^22^

### Limitations:

This is a single center retrospective study with a relatively small sample size and may be prone to selection bias. Additionally, neither group was randomly assigned, and baseline information may be imbalanced and biased. There were only few observation indicators, and a limited follow-up period of six months. The impact of the combination of titanium locking plate and threaded rivet surgery on the long-term functional recovery of patients was not analyzed. Further higher quality research is needed to verify our conclusion.

## CONCLUSION

Adopting a combination of titanium locking plates and SA repair to treat AF can increase active range of motion and muscle strength of the ankle joint in plantar flexion and dorsiflexion states, reduce the medial clear space and talar tilt angle. In addition, it is beneficial for improving the ankle joint functional recovery, improving patients’ quality of life, and reducing the incidence of complications.

### Authors’ Contribution:

**ZH:** Contributed to the study design, questionnaire design, data interpretation, and provided feedback through critical manuscript review. **ZH and MC:** Contributed to the study concept, study design, literature search, data collection, analysis, and interpretation. **ZH, MC and ZY:** Contributed to the literature search and manuscript writing. All authors have read the final version and are responsible and accountable for the accuracy and integrity of the work.
